# Plasticized Mechanical Recycled PLA Films Reinforced with Microbial Cellulose Particles Obtained from Kombucha Fermented in Yerba Mate Waste

**DOI:** 10.3390/polym15020285

**Published:** 2023-01-05

**Authors:** Ángel Agüero, Esther Corral Perianes, Sara Soledad Abarca de las Muelas, Diego Lascano, María del Mar de la Fuente García-Soto, Mercedes Ana Peltzer, Rafael Balart, Marina Patricia Arrieta

**Affiliations:** 1 Instituto de Tecnología de Materiales (ITM), Universidad Politécnica de Valencia (UPV), Plaza Ferrándiz y Carbonell 1, 03801 Alcoy, Spain; 2 Departamento de Ingeniería Química Industrial y del Medio Ambiente, Escuela Técnica Superior de Ingenieros Industriales, Universidad Politécnica de Madrid (ETSII-UPM), Calle José Gutiérrez Abascal 2, 28006 Madrid, Spain; 3 Grupo de Investigación: Tecnologías Ambientales y Recursos Industriales (TARIndustrial), 20006 Madrid, Spain; 4 Laboratory of Obtention, Modification, Characterization, and Evaluation of Materials (LOMCEM), Department of Science and Technology, University of Quilmes, Bernal B1876BXD, Argentina; 5 National Scientific and Technical Research Council (CONICET), Buenos Aires C1425FQB, Argentina; 6 Grupo de Investigación: Polímeros, Caracterización y Aplicaciones (POLCA), 28006 Madrid, Spain

**Keywords:** PLA, cellulose, yerba mate, kombucha, food packaging

## Abstract

In this study, yerba mate waste (YMW) was used to produce a kombucha beverage, and the obtained microbial cellulose produced as a byproduct (KMW) was used to reinforce a mechanically recycled poly(lactic acid) (r-PLA) matrix. Microbial cellulosic particles were also produced in pristine yerba mate for comparison (KMN). To simulate the revalorization of the industrial PLA products rejected during the production line, PLA was subjected to three extrusion cycles, and the resultant pellets (r3-PLA) were then plasticized with 15 wt.% of acetyl tributyl citrate ester (ATBC) to obtain optically transparent and flexible films by the solvent casting method. The plasticized r3-PLA-ATBC matrix was then loaded with KMW and KMN in 1 and 3 wt.%. The use of plasticizer allowed a good dispersion of microbial cellulose particles into the r3-PLA matrix, allowing us to obtain flexible and transparent films which showed good structural and mechanical performance. Additionally, the obtained films showed antioxidant properties, as was proven by release analyses conducted in direct contact with a fatty food simulant. The results suggest the potential interest of these recycled and biobased materials, which are obtained from the revalorization of food waste, for their industrial application in food packaging and agricultural films.

## 1. Introduction

Biobased and biodegradable polymers have gained attention for food packaging applications in order to reduce the consumption of non-renewable resources and prevent the accumulation of plastic waste in the environment. Among other biopolymers, poly(lactic acid) has emerged in the market as the most used biobased and biodegradable plastic due to its many advantages, such as its environmentally benign characteristics, availability in the market at a competitive cost, ease of processing by means of the current existing processing technologies for petrol-based thermoplastics (i.e., extrusion, injection molding, etc.), high transparency, and inherent biodegradability [1,2]. However, PLA also presents some disadvantages for film production which hinder its industrial exploitation in the food packaging or agricultural sectors, such as its sensitivity to thermal degradation [2], poor barrier performance [3], and inherently brittle nature [4]. Its degradability in the environment requires specific conditions (compost medium at 58 °C, a pH around 7.5, relative humidity of 60%, a C/N relationship between 20:1 and 40:1, and proper aeration) to be met, even for short periods of time [5]. Moreover, the model of the linear economy generates high levels of plastic waste and creates a dependence between economic development and the entry of new, virgin plastics into the system [6]. Therefore, the use of recycled PLA for film for food packaging or agricultural applications is gaining interest [7,8]. Cosate de Andrade et al. [8] compared the chemical recycling and mechanical recycling of PLA, and concluded that mechanical recycling generates less impact than chemical recycling due to the fact that the mechanically recycled polymers are produced using lower energy and fewer inputs than other destinations. However, bioplastic consumption is currently still low, and they can be considered contaminants in plastic recycling streams due to the fact that they can affect the mechanical performance of well-implemented mechanical recycling processes of other plastics, such as polyethylene terephthalate (PET), polypropylene (PP), and polystyrene (PS) [9,10,11]. Moreover, although the European Commission promotes the increase of recycled plastic in food packaging as an essential prerequisite to its strategy to introduce recycled plastics in a circular economy, the current legislation does not allow the direct use of recycled plastics coming from recycled streams for food contact materials. This is because those recycling processes originate from waste, and the legislation establishes strict requirements concerning food safety (the transfer of substances that may affect human health, or quality of the food, and microbiological safety) [12]. In this context, during the industrial production of plastic products, several parts are produced with defects, rejected from the production line, and then discarded. These rejected parts can be reprocessed and used to produce recycled pellets that do not come from waste streams and are of well-known origin. In a previous work, PLA was reprocessed up to six times, and it was observed that the main losses took place when PLA was subjected to more than four reprocessing cycles, while low degradation was found between one and three reprocessing cycles [13]. However, due to PLA’s high sensitivity to hydrolysis of its ester groups at the industrial processing conditions, such as melt extrusion, the obtained recycled PLA-based products show a decrease in the polymer chain length and, thus, show lower-quality performance than PLA-based products produced with virgin PLA [14]. This is why the use of reinforcing fillers with antioxidant activity as additives have gained interest for the purpose of protecting the polymeric matrix from thermal degradation and increasing the mechanical resistance of mechanically recycled PLA [7].

Another industrial sector that generates a large amount of waste and can be introduced in the food packaging sector for the preparation of high-tech composites and/or nanocomposites is the food industry [15]. The kombucha beverage is a popular probiotic beverage typically produced by fermenting sugared tea with a symbiotic community of bacteria and yeast (SCOBY) that involves cooperative and competitive interactions [16]. While yeasts produce invertase, which releases monosaccharides to media accessible to any microbe as a carbon source, bacteria rapidly metabolize released sugars and produce organic acids that acidify the media [16]. Meanwhile, the reduction in monosaccharides increases the frequency of the invertase-producing yeast, and the ethanol produced by yeast stimulates the bacterial cellulose synthase mechanism to produce a cellulose film at the surface that acts as a physical barrier to protect from external competitors [16]. The cellulosic film is a byproduct in the kombucha tea industry, but it is very interesting for the plastic industry. Kombucha tea has been fermented in several sugared infusions (i.e., black tea, green tea, yerba mate, etc.) [16,17,18]. The antioxidant activity of microbial cellulose obtained from the kombucha fermentation is directly related to the high amount of bioactive compounds in the infusion used for its fermentation, such as phenolics, tannins, catechins, flavonoids, etc., which are decomposed into their simpler forms during the kombucha fermentation process [17]. In fact, it has been observed that the cellulose obtained from kombucha fermented in sugared infusions of yerba mate possesses high antioxidant activity [16]. Yerba mate (*Ilex paraguariensis*, Saint Hilaire) is a tree from the subtropical region of South America that grows in a limited zone within Argentina, Brazil, and Paraguay, where it has an important commercial purpose due to the high consumption of dried yerba mate leaves in the form of infusion, which is known as “mate” [15,19]. Its high consumption leads to a high amount of yerba mate being wasted without any kind of revalorization [15]. For instance, in 2020, the consumption of yerba mate in Argentina was over 310 million kg [20]. Thus, in this work, kombucha SCOBY was fermented in yerba mate waste.

Among other plasticizers, citrate esters such as acetyl(tributyl citrate) (ATBC) have been proven to be very effective PLA plasticizers, and are accepted for food contact applications [21]. The miscibility between PLA and ATBC has been associated with the similarity in their solubility parameters (*δ*) that of PLA being between 19.5 MPa^1/2^ and 20.5 MPa^1/2^ [2], while that of ATBC is 20.2 MPa^1/2^ [22]. Likewise, to produce polymers by a solvent casting method, the selection of an effective solvent is also on the basis of a similar solubility parameter to that of the polymer. In this sense, chloroform (*δ* = 19 MPa^1/2^) is widely used to dissolve PLA [23].

The main objective of the present research was to obtain sustainable and active films from the revalorization of plastic and food industry waste. The materials were prepared based on mechanically recycled PLA and cellulosic particles extracted from kombucha fermented in yerba mate waste. Thus, virgin PLA was subjected to three reprocessing extrusion cycles (r3-PLA) to simulate the revalorization of industrial PLA products rejected during the production line. Three reprocessing cycles were selected, since in a previous work, it was observed that between one and three reprocessing cycles, low PLA degradation occurs [13]. The decrease in the polymer chain length due to the three reprocessing cycles was investigated by measurement of the viscosity-molecular weight. On the other hand, yerba mate waste was used to obtain the sugared infusion to produce kombucha beverage from kombucha SCOBY, while the cellulosic by-product formed during its production was used to produce cellulosic particles with antioxidant activity (KMW). Another kombucha SCOBY was fermented in a sugared infusion of new yerba mate, and the cellulosic particles obtained were studied for comparison (KMN). Both particles, namely kombucha mate waste (KMW) and kombucha mate new (KMN), were used to reinforce plasticized, mechanically recycled r3-PLA with 15 wt.% of ATBC. Two reinforcing amounts were used, namely 3 wt.% and 5 wt.%, and the obtained films were characterized in terms of transparency, barrier performance against water and UV light, thermal stability, crystallization behavior, surface wettability, and mechanical performance in order to obtain information regarding the possibility of using these films as antioxidant food contact materials, such as food packaging or in the agro-industrial field.

## 2. Materials and Methods

### 2.1. Materials

PLA commercial-grade IngeoTM 2003D with a density of 1.24 g·cm^−3^ and a melt flow index (MFI) of 6 g/10 min (measured at 210 °C and with a load of 2.16 kg) was supplied by Natureworks (Minnetonka, MN, USA). Acetyl tributyl citrate (ATBC) (98% purity, Mw = 402 g mol^−1^, and Tm = −80 °C), chloroform (CHCl_3_, *δ* = 19 MPa^1/2^), and 2,2-diphenyl-1-picrylhydrazyl (DPPH) 95% free radical were supplied by Sigma Aldrich (Madrid, Spain). The pristine yerba mate (Taragüi, Virasoro, Argentina) was used as is and called YMN, while the yerba mate waste was obtained from the residue of mate infusion after our consumption and called YMW.

### 2.2. Processing of Kombucha to Obtain Cellulosic Particles from Yerba Mate Waste

**Figure 1:**
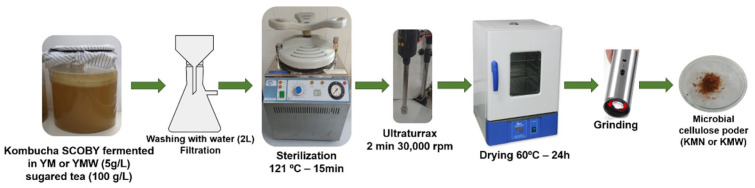
Schematic representation of the microbial cellulose (KMN and/or KMW) production from kombucha fermented in YM or YMW.

The native culture of kombucha was provided by Teresa Carles Manufacturing S. L. (Barcelona, Spain), and was used as the starter culture and inoculum for a new batch of kombucha fermented in an infusion of yerba mate (5 g/L) and sucrose (100 g/L). KMW was obtained from the fermentation of one kombucha SCOBY from that batch in a 2.5 L sugared infusion prepared either with 15 g of yerba mate (YMN) and/or yerba mate waste (YMW), 300 g of sucrose, and 500 mL of stock culture, which was maintained at static conditions at 22 ± 2 °C and then covered with a textile cloth for 30 days. A new floating disc was produced, and it was recovered, washed with distilled water, filtered off, and further sterilized at 121 °C and 101 kPa for 15 min in a steam autoclave. The disc was then homogenized by ultraturax at 30,000 rpm for two minutes (4 cycles of 30 s) and dried at 60 °C for 24 h. The dry matter, determined by drying at 105 °C until a constant weight was reached, showed a yield of ca. 1.3 ± 0.1%, in accordance with previous reported works [16]. Then, the obtained cellulosic paper was ground to obtain a powder and further sieved (500 μm). In Figure 1 the wall process to obtain either KMN or KMW from the SCOBY fermented in YM or YMW and convert it to the powder able to be processed by melt extrusion is schematically represented.

### 2.3. Processing and Reprocessing of PLA

To obtain reprocessed PLA (r3-PLA), PLA pellets were previously dried overnight to remove the residual moisture at 60 °C for 4 h in an air-circulating oven. The PLA pellets were processed 3 times in a twin-screw co-rotating extruder with a screw diameter of 30 mm, supplied by Construcciones Mecanicas Dupra, S.L. (Alicante, Spain), at a screw speed of around 22 rpm and using a temperature profile of 180 °C (feeding hopper), 185 °C, 190 °C, and 195 °C (extrusion die), on the basis of previous work [13]. After the extrusion process, the strands were cooled in air and then pelletized using an air-knife unit. They were subsequently subjected to an additional processing cycle under the same conditions, up to three times.

The capillary viscosity of virgin PLA and r3-PLA pellets was measured with a Ubbelohde viscometer (type 1C). Both pellets were diluted in CHCl_3_ and the measurements were conducted at 25 °C using a water bath and a home-made 3D printed viscosimeter support. At least four concentrations were used. The intrinsic viscosity [η] of PLA and r3-PLA was determined to estimate the viscosity molecular weight by means of the Mark–Houwink relation (Equation (1)).

(1) $$\left[\eta \right]=K\times M_{v}^{a}$$

where *K* and *a*, for PLA, are 1.53 × 10^−2^ and 0.759, respectively [24].

### 2.4. Films Preparation

KMN- and KMW-loaded r3-PLA-ATB-based materials were processed into thin films by the solvent casting method. For this purpose, 0.6 g of reprocessed PLA pellets (r3-PLA) were dissolved in 45 mL of CHCl_3_ under continuous stirring at 1000 rpm at room temperature. ATBC was then added at 15 wt.% with respect to the polymeric matrix, on the basis of previous works [15,22,25], and named r3-PLA-ATBC. For the development of composites, the plasticized PLA films (r3PLA-ATBC) were then loaded either with kombucha mate waste (KMW) or kombucha mate new (KMN) in 1 wt.% and 3 wt.%, with respect to the r3-PLA-ATBC polymeric blend, and all films were prepared by the solvent casting method. Each suspension was cast onto a 50 mm-diameter glass mold, and then CHCl_3_ was allowed to evaporate at 40 °C for 48 h in an oven. The obtained films are summarized in Table 1. They were dried under a vacuum to complete the drying process, ensuring the complete elimination of the solvent for about 10 h at 40 °C, prior to being characterized. 

### 2.5. Characterization of the Films

#### 2.5.1. UV-Visible Measurements

The transmittance of the obtained films was measured in the 800–250 nm region using a UV-Visible spectrophotometer Varian Cary 1E UV-Vis (Varian, Palo Alto, CA, USA) at a scanning speed of 400 nm/min. The overall transmittance in the visible region was calculated following the ISO 13468 standard.

#### 2.5.2. Scanning Electron Microscopy

The microstructures of films’ cross-sections were observed by field emission scanning electron microscopy (FESEM) by means of a ZEISS ULTRA 55 microscope from Oxford Instruments (Abingdon, UK). The film samples were previously frozen in liquid N_2_, cryofractured, and sputtered with a thin layer of gold and palladium alloy in an EMITECH sputter coating, SC7620, from Quorum Technologies, Ltd. (East Sussex, UK) to achieve a conductive surface. Then, the film samples were observed with an accelerating voltage of 2 kV. Images were taken at 10,000× magnification.

#### 2.5.3. Differential Scanning Calorimetry

Differential scanning calorimetry (DSC) analyses were conducted in a Mettler-Toledo model 821 DSC (Schwerzenbach, Switzerland). The DSC thermal cycles were carried out under a nitrogen atmosphere. The first heating DSC scan was conducted from 30 °C to 200 °C at a rate of 10 °C/min, with the main objective of eliminating the thermal history. Then, the samples were cooled down to −50 °C at a rate of 10 °C/min. Finally, the second heating DSC scan was carried out from −50 °C to 300 °C at a rate of 10 °C/min. The degree of crystallinity ($\chi_{c}$), obtained from the DSC thermograms, was calculated by Equation (2).

(2) $$\chi_{c}=\frac{\Delta H_{m}-\Delta H_{cc}}{\Delta H_{m}^{0}}\cdot \frac{1}{W_{PLA}}100$$

where Δ*H_m_* is the melting enthalpy, Δ*H_cc_* is the cold crystallization enthalpy, $\Delta H_{m}^{0}$ is the melting heat associated with pure crystalline PLA (93 J g^−1^) [26], and *W_PLA_* is the weight fraction of PLA in the blend formulation.

#### 2.5.4. Thermogravimetric Analysis

Dynamic thermogravimetric analyses were conducted in a TA Instruments TGA2050 thermobalance (TA Instruments, New Castle, DE, USA). For each measurement, around 10 mg of films were placed in a platinum crucible and heated from 30 to 800 °C at 10 °C/min, under a nitrogen atmosphere.

Isothermal thermogravimetric analyses were also conducted in a TGA/SDTA 851 thermobalance from Mettler-Toledo (Schwerzenbach, Switzerland). For each measurement, around 10 mg of films were heated at 180 °C for 20 min.

#### 2.5.5. Tensile Test Measurements

The mechanical properties were evaluated by means of tensile test measurements using a Shimadzu AGS-X 100 N universal tensile testing machine (Shimadzu Corpo-194 ration, Kyoto, Japan) equipped with a 100 N load cell, with an initial length of 30 mm and a crosshead speed of 10 mm min^−1^. Dog-bone samples were prepared by a JBA electrohydraulic cutter (Instruments J. Bot SA) for tensile specimen 1BB, according to ISO 527-2. The Young modulus (E), tensile strength (TS), and average percentage elongation at break (ε%) were calculated from the obtained stress–strain curves, and the media of at least five specimens were reported.

#### 2.5.6. Static Contact Angle Measurements

Surface wettability of the films was studied through static water contact angle (WCA) measurements by using a standard goniometer (EasyDrop-FM140, KRÜSS GmbH, Hamburg, Germany) equipped with a camera and Drop Shape Analysis SW21; DSA1 software. Drops of ~15 μL distiller water were placed onto the films’ surfaces with the aid of a syringe, and approximately ten contact angle measurements were taken for each sample, with the films in random positions. 

#### 2.5.7. Water Vapor Transmission Rate

The water vapor transmission rate (WVTR) measurements of the films were determined by gravimetry, using silica gel as a desiccant agent. Films were placed in permeability cups with an exposed area (A) of 10 cm^2^, filled with 2 g of previously dried silica gel, and further placed in a desiccator at 23 ± 1 °C with a saturated KNO_3_ solution, obtaining a relative humidity of 85 ± 4%. The cups were weighed every hour for 7 h, and then again after 24 h. The mass increase in the cups was plotted against time, with slope *n*. WVTR (g/day cm^2^) was determined through Equation (3):

(3) $$\mathrm{WVTR}=\frac{\;n}{A}$$

Because the water vapor transmission is dependent on the film thickness, the WVTR values were normalized to 100 μm [27].

#### 2.5.8. Specific Migration Test and Antioxidant Activity

Double-sided total immersion migration tests were performed by total immersion of films in a glass vial containing a fatty food simulant (Simulant D1 = ethanol 50% *v/v*) at 40 °C for 10 days (area-to-volume ratio = 6 dm^2^/L) [28]. After 10 days, films were removed and the food simulant was used to determine their antioxidant ability, which was measured by determination of the radical scavenging activity (RSA) through the DPPH method. This was accomplished by the determining the reduction in the absorbance at 517 nm by means of a UV-Vis Varian Cary spectrophotometer. The radical scavenging activity (RSA) was determined using Equation (4).

(4) $$\mathrm{RSA}\;\left(\%\right)=\frac{A_{control}-A_{sample}}{A_{control}}\times 100\%\;$$

where *A_control_* is the absorbance of 2,2-difenil-1-picrylhydrazyl (DPPH) in ethanolic solution and *A_sample_* the absorbance of DPPH after 15 min in contact with each food simulant sample.

## 3. Results

### 3.1. Reprocessed PLA Characterization

The materials developed herein were prepared with mechanically recycled PLA, which was processed three times by melt extrusion using a temperature profile from feeding to hopper of 180 °C, 185 °C, 190 °C, and 195 °C, based on previous work [13], to simulate the revalorization of industrial waste produced during the production line, in which some parts are rejected. The viscosity–molecular weight (*M_v_*) relationship of the reprocessed PLA (3r-PLA) and PLA pellets was determined in order to obtain insights into the degradation of the polymeric matrix as a consequence of the reprocessing procedure. The obtained results of the estimated *M_v_* of PLA and r3-PLA were 181,770 ± 3370 g/mol and 115,410 ± 5080 g/mol, respectively (a reduction of around 36%). A reduction in the intrinsic viscosity ([η]) has already been reported in PLA samples subjected to a simulated mechanical recycling process in which one melt extrusion reprocessing cycle was applied, showing a reduction of around 14% with respect to samples prepared with virgin PLA [29]. In the present work, a reduction in the intrinsic viscosity ([η]) due to the three reprocessing melt extrusion cycles was around 30%, leading to the aforementioned reduction in the *M_v_*. This reduction in the *M_v_* is due to chain scission, produced by thermal degradation during each thermal processing cycle as a consequence of a hydrolysis process which is augmented by the heating [13].

### 3.2. UV-Visible Measurements

**Figure 2:**
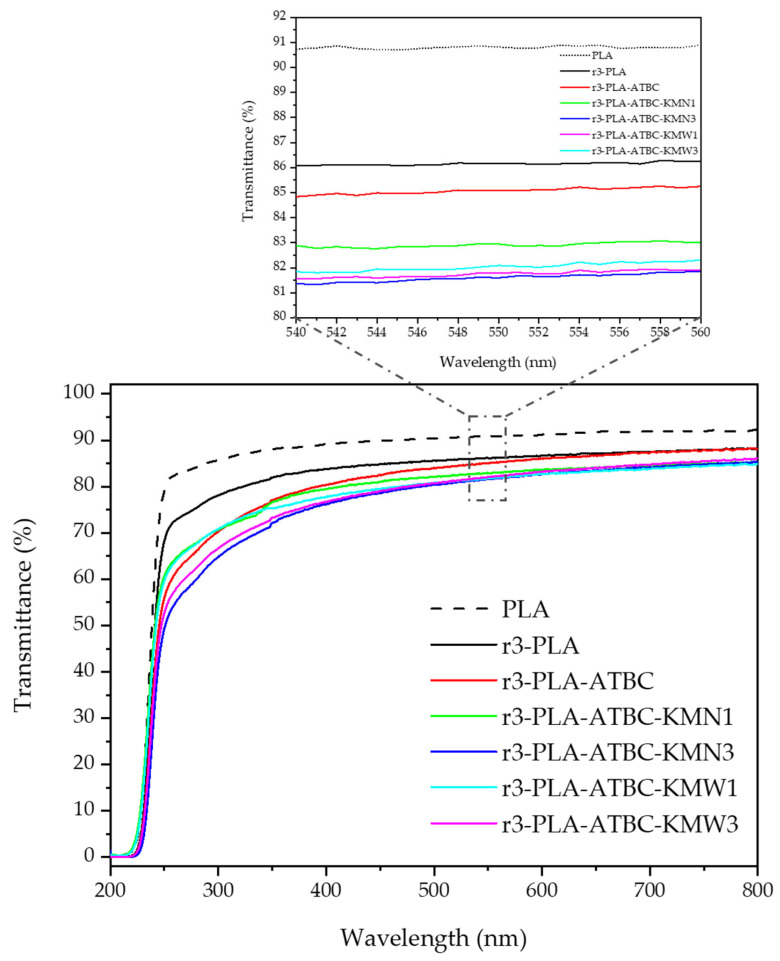
UV-vis spectra of films 200–800 nm and zoom image 540–560 nm.

The transmittance of the obtained films was measured by means of a UV-Visible spectrophotometer, and the absorption spectra of the films are shown in Figure 2. Neat PLA film was also analyzed for comparison. From the spectra, it could be seen that although all formulations based on recycled PLA (r3-PLA) resulted in less transparent materials than PLA, they were mostly transparent in the visible region of the spectra (400–700 nm) allowing the films to be seen through, which is one of the most important requirements for food packaging due to consumers’ acceptance [30]. It is also very important that films intended for agricultural applications should not only protect crops, but also permit the photosynthesis process to occur [1,31]. Among reprocessed films, r3-PLA film was the most transparent film, showing the highest transmission along the visible region of the spectra (400–700 nm). The incorporation of ATBC slightly affected the transparency of r3-PLA, as was already observed with the addition of ATBC to a virgin PLA matrix [22]. The transparency was slightly reduced with the incorporation of KMN and/or KMW. Absorption measurements were conducted in the range of 540–560 nm (see zoom image in Figure 2) of the visible region of the spectra, and it can be seen that the materials resulted as highly transparent (between 81% and 87% of transmittance). 

When comparing r3-PLA with neat PLA film, a slight UV-light absorption in the 260 to 290 nm region can be observed in the 3r-PLA sample, which has already been observed in recycled PLA [7,14]. It has been reported that recycled PLA leads to a reduction in the UV light transmission in the 260 to 290 nm region of the spectrum, ascribed to the formation of –COOH chain end groups in PLA as a consequence of the chain scission (carbonyl carbon-oxygen bond cleavage) during thermal processing [14]. Nevertheless, it should be highlighted that the UV light transmission reduction is less marked than in the post-consumer, mechanically recycled PLA bottles studied by Chariyachotilert et al. They observed higher UV light transmission reduction, which can be related not only to the thermal degradation during reprocessing, but also with the degradation of PLA products during service, as well as under the conditions typically used for cleaning PET (85 °C, 1 wt.% NaOH and 0.3 wt.% Triton^®^ X-100 surfactant for 15 min) [14]. In this sense, in a previous work, Agüero et al. studied the mechanical recyclability of injected molded PLA parts in more depth, performing between one and six reprocessing melt extrusion cycles, and showed that low degradation takes place between one and three reprocessing cycles in [13]. Thus, this means that less degradation had taken place after three reprocessed melt extrusion cycles than in post-consumed, washed, and further reprocessed PLA, highlighting the viability of mechanical recyclability of rejected PLA parts from the production line.

### 3.3. Scanning Electron Microscopy

**Figure 3:**
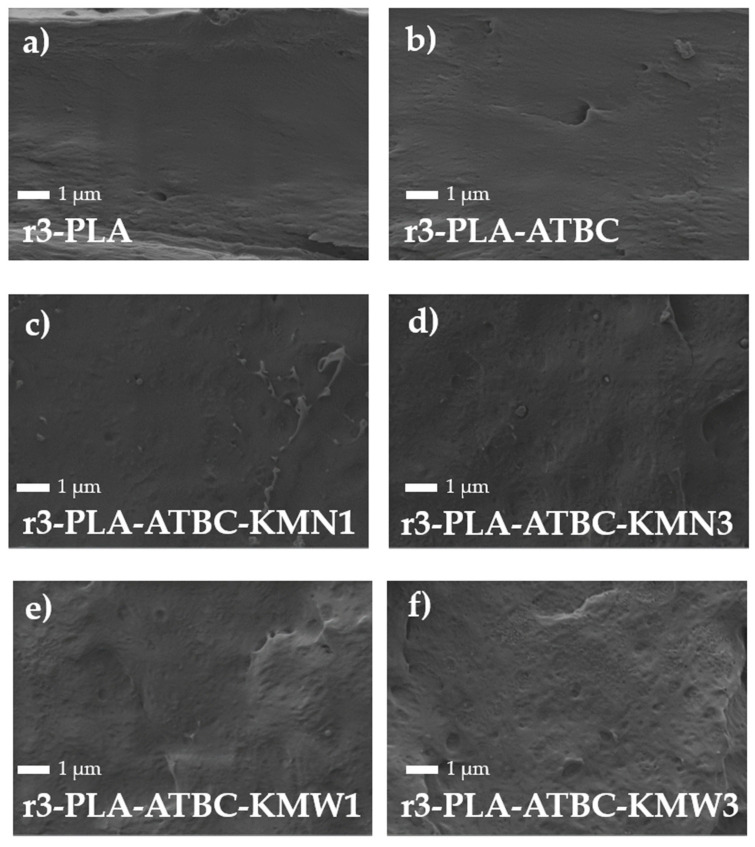
FE-SEM observations at 10,000× of 3r-PLA-ATBC based films: (**a**) r3-PLA, (**b**) r3-PLA-ATBC, (**c**) r3-PLA-ATBC-KMN1, (**d**) r3-PLA-ATBC-KMW1, (**e**) 3r-PLA-ATBC-KMN3, and (**f**) r3-PLA-ATBC-KMW3.

FESEM investigations were conducted to study the microstructure of the films, and the micrographs of the cross-fractured surface are shown in Figure 3. The r3-PLA film (Figure 3a) showed the typical regular and smooth fracture of PLA films based on semi-crystalline virgin PLA [9,32]. An increased ductile fracture was observed in r3-PLA-ATBC film (Figure 3b), with more plastic behavior and no apparent phase separation, demonstrating the plasticizing effect of ATBC on the reprocessed PLA matrix. The ternary composites for both formulations with 1 wt.% (Figure 3c,e) and those with 3 wt.% (Figure 3d,f), showed a uniform dispersion of both KMN and KMW into the r3-PLA matrix. In the case of the higher reinforcing amount used here (3 wt.%) (Figure 3d,f), it seems that there was an increase in surface roughness. However, the formulations had very similar surface patterns to those reinforced with lower amounts of kombucha particles (1 wt.%), suggesting that cellulosic particles are well-distributed in the reprocessed PLA matrix. It has been observed that plasticizers such as ATBC improve the dispersion of cellulosic particles into the PLA matrix [25].

### 3.4. Differential Scanning Calorimetry

**Figure 4:**
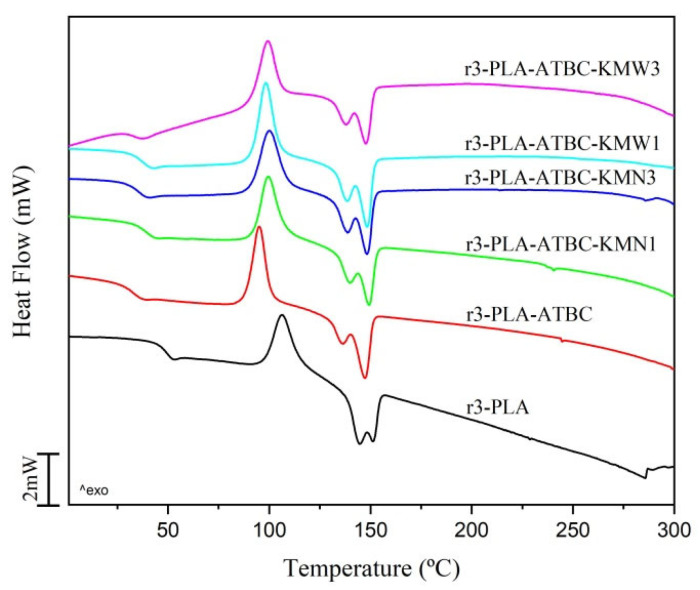
DSC second heating scan of 3r-PLA-ATBC-based films.

DCS analysis was conducted and used to investigate the glass transition (T*_g_*), cold crystallization (T*_cc_*), melting temperatures (T*_m_*), and crystallinity (*χ_c_*) of plasticized 3r-PLA-ATBC films, and the obtained DSC curves are shown in Figure 4 while the obtained results are summarized in Table 2. The r3-PLA film showed the T*_g_* at a lower value than the PLA samples which were processed three times by melt extrusion and further processed by injection molding (T*_g_* = 64 °C [13]), due to the presence of a residual solvent, as was demonstrated by Yang et al. Their study compared PLA-based composites processed by extrusion with those processed by solvent casting method, and concluded that limited variations in the DSC parameters were observed for samples processed with the two different processing techniques (melt extrusion and solvent casting method) [33]. In the r3-PLA film, a cold crystallization peak appeared which was not present in the virgin PLA pellet [13], and this has been related to the fact that the shorter PLA chains formed during the reprocessing cycles, such as oligomers, showed higher mobility levels and promoted the crystallization of PLA [13,29]. In fact, it has been observed that PLA plasticized with oligomeric lactic acid (OLA) showed a reduction in cold crystallization temperature, which was further reduced by increasing the OLA content [34,35]. Similarly, the incorporation of the ATBC plasticizer produced a decrease in the T*_g_* and T*_cc_*, as well as in the T*_m_*, which is ascribed to the ability of the ATBC plasticizer to increase the free volume between the polymer chains. Accordingly, their mobility was also decreased, enhancing the slow crystallization rate [22,36]. On the other side, the combination of the ATBC plasticizer and the microbial cellulose particles onto a plasticized 3r-PLA matrix produced higher T*_g_* and T*_cc_* values and higher crystallinity degrees, suggesting that the segmental motion of PLA matrix may have been affected by the presence of KMN and KMW [33]. Moreover, the synergic effect on the crystallization of PLA as a consequence of a potential nucleating agent in a presence of citrate ester plasticizers has been already reported [25,37]. The DSC thermograms show a double melting behavior, which has already been observed in mechanically recycled PLA [38,39] and PLA plasticized with OLA [34]. This behavior, in PLA-based materials, is ascribed to the presence of different crystalline structures with different levels of perfection and thermodynamic stability. The melt PLA crystallizes at temperatures higher than 120 °C in an ordered form (α form) [40]. In the present work, all samples were crystallized at temperatures below 120 °C. This is related to the ability of shorter polymer chains (oligomers), produced as a consequence of the PLA degradation during reprocessing steps, to promote the aforementioned crystallization of PLA. The reduction in the cold crystallization temperature was particularly marked in plasticized 3r-PLA-ATBC samples. When PLA crystallized below 110 °C, less stable crystals appeared, known as α′ crystals [40]. From the cold crystallization peak in the DSC thermogram, it can be observed that disorder (crystals with α′ form) to order (crystals with α form) phase transition took place, suggesting that a great fraction of the polymer was in an amorphous state due to the DSC cooling scan applied, as this was already observed in plasticized PLA-ATBC samples [15,41]. An increase in the T*_cc_* values of KMN- and KMW-loaded films was observed with respect to the r3-PLA-ATBC film, suggesting that somewhat fewer disordered crystals (α′) are present in composite materials. A different crystallization degree was observed for PLA-ATBC-KMN-based films with respect to PLA-ATBC-KMW-based films. A higher crystallinity degree was found for those particles obtained from the fermentation of kombucha in yerba mate waste (KMW), and could be directly related to the superior dispersion of KMW particles into the plasticized PLA-ATBC matrix, which are able to promote a higher nucleation effect [25].

### 3.5. Thermogravimetric Analysis

**Figure 5:**
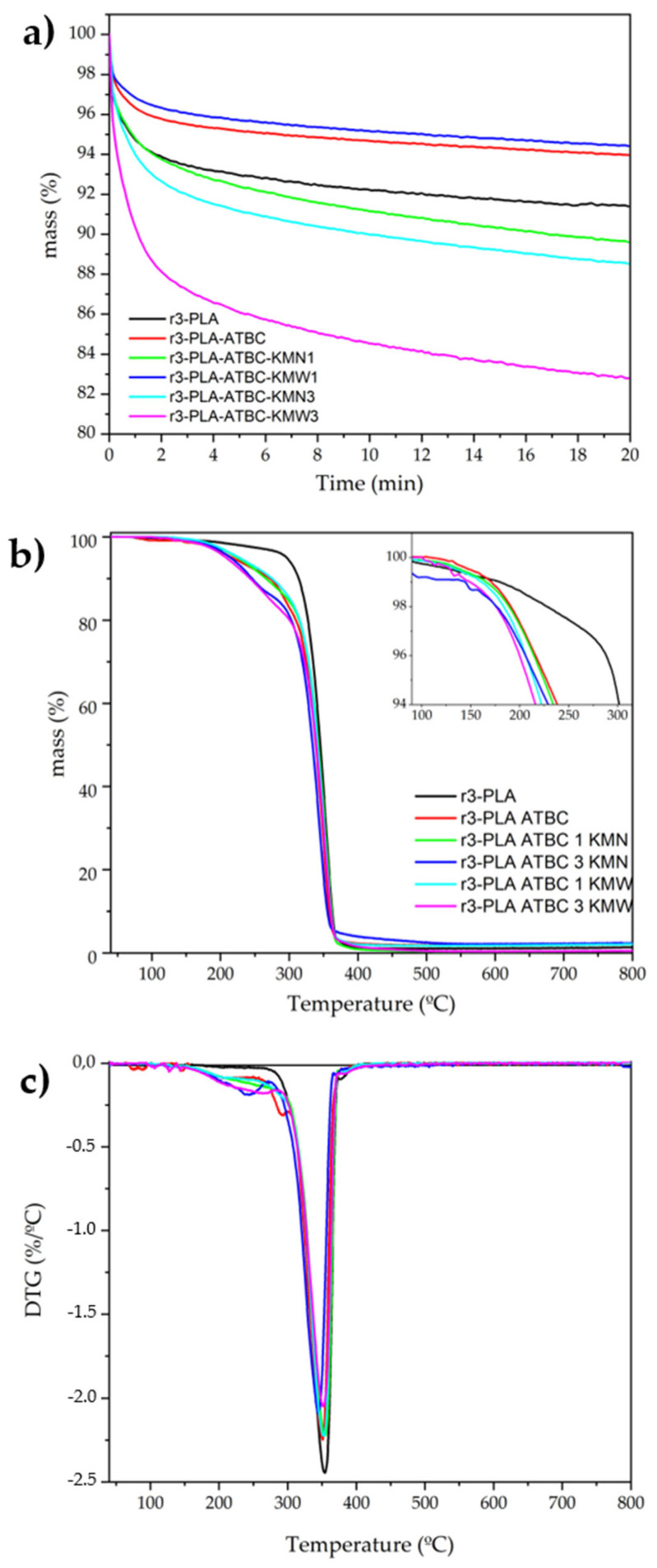
(**a**) Isothermal TGA analysis at 180 °C and Dynamic TGA (**b**) and DTG (**c**) of 3r-PLA-ATBC-based films.

The thermal stability of the materials was studied under isothermal mode at 180 °C to ensure enough thermal stability for the typical melt-processing temperature of PLA (Figure 5a). During the first minute under TGA isothermal conditions, films experienced a quick weight loss, probably due to the evaporation of the remaining chloroform. Then, all the materials showed a mass loss of around 1 or 2% in 10 min, which allows enough time to process the PLA-based materials. 3r-PLA-ATBC-KMW1 showed very similar thermal stability to 3r-PLA-ATBC, whereas 3r-PLA-ATBC-KMW3 presented less thermal stability. This could be related to the fact that when higher amounts than 3 wt.% of KMW reinforce 3r-PLA, some part of the PLA matrix is non-stabilized due to the deficient particle dispersion. Nevertheless, it should be highlighted that all the materials showed enough thermal stability for melt extrusion purposes.

The thermal degradation parameters obtained by TGA are described in Table 3. Plasticization of PLA produced a decrease in T_5%_ and T_10%_, due to the decomposition of the plasticizer [42,43], and a slight decrease in T_max_ was observed, since the plasticization could make the polymer chains available to thermal degradation, as was already observed on plasticized PLA with citrate esters [15,22,30]. The addition of 1% of KM increased T_5%_ and T_10%_ in the case of KMW, compared with plasticized r3-PLA. However, the addition of 3 wt.% of KM decreased T_5%_ and T_10%_ due to the low thermal stability of bacterial cellulose [44]. Regarding T_max,_ it seemed that this value was enhanced by the addition of KMW and KMN at 1 wt.%, since the value was close to the unplasticized r3-PLA film, but the addition of a higher amount of KM showed a significant decrease for 3 wt.% KMN. However, no modifications were observed for KMW. An overall conclusion for this study is that the addition of KMW enhanced the thermal properties of r3-PLA-ABTC better than the KMN. This could be due to the higher crystallinity of r3-PLA-ABTC-KMW1 and r3-PLA-ABTC-KMW3 composites, as shown by the DSC results.

Figure 5 also shows the TGA (Figure 5b) and DTG (Figure 5c) curves. Thermal degradation of the composites presented two steps of degradation. Firstly, the evaporation/degradation of the plasticizer overlaps with the initial degradation of KM, then in the second step of degradation, a PLA matrix was observed. For samples with higher concentrations of KM, a higher percentage of the mass was lost in the first step, confirming that in this event, the KM was starting to degrade. It is important to note that the composites were thermally stable at the processing temperatures usually used for PLA.

### 3.6. Tensile Test

**Figure 6:**
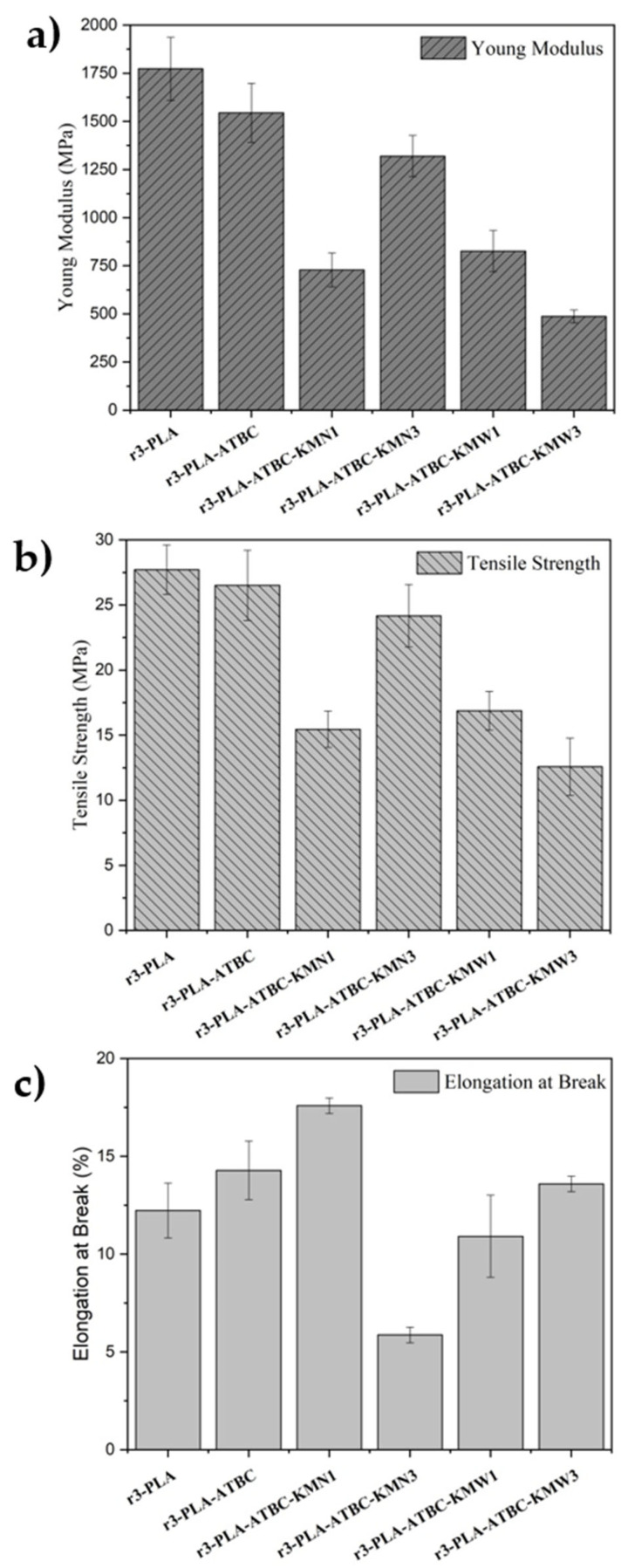
Tensile test measurements of 3r-PLA-ATBC-based films: (**a**) Young modulus, (**b**) Tensile Strength and (**c**) Elongation at break.

The mechanical performance of the r3-PLA and plasticized r3-PLA based films was analyzed by tensile test, the results of which, in terms of Young Modulus (MPa), Tensile Strength (MPa), and Elongation at Break (%), are represented in Figure 6. Firstly, it is worth noting the decreased tensile parameters obtained for the r3-PLA films analyzed herein, which were processed by solvent casting, compared with in other works centered around PLA samples, which used melt extrusion [13]. As was mentioned previously, this effect of the processing condition on the mechanical behavior of PLA films has been studied by Yang et al. [33], who related this reduction in the tensile values, especially in terms of modulus, to the presence of captured residual chloroform, which acts as a plasticizer. Otherwise, with the addition of ATBC (r3-PLA-ATBC), an enhanced in ductility was observed, as was expected due to the proven effectiveness of this citrate ester as a PLA plasticizer [22,25,37,45]. Specifically, r3-PLA-ATBC showed a notable modulus reduction with respect to r3-PLA, from ~1750 MPa to ~1550 MPa, as well as an increment in the elongation at break from 12% to 14%, while a very small drop in the tensile strength was observed.

Regarding the addition of KM, at low content (1 wt.%), both KMN and KMW induce a very similar effect on the modulus and tensile strength, showing r3-PLA-ATBC-KMN1 and r3-PLA-ATBC-KMN1 values around 750–850 MPa and 15–17 MPa, respectively, for these parameters. In composite materials, when the particle dispersion is not homogenous enough, the transfer load does not occur appropriately, which caused a reduction in modulus and strength. This drawback of the introduction of cellulose-derived particles in PLA films has already been reported by other authors [46]. However, a significant difference between KMN and KMW was observed for the results of the elongation at break. In this sense, r3-PLA-ATBC-KMN1 showed a slight enhancement with respect to unloaded r3-PLA-ATBC (up to 17%) while the results of the r3-PLA-ATBC-KMW1 samples were closer to the non-plasticized r3-PLA. This difference in terms of ductility is directly correlated with the crystallinity values obtained in the thermal analysis. When the KM content was increased, the r3-PLA-ATBC film loaded with 3 wt.% of KMN showed a somewhat higher modulus and tensile strength, approaching the r3-PLA-ATBC, while the elongation at break was shown to be lower. Lignocellulosic and cellulosic materials have been widely studied as fillers of PLA-based composites due to their high weight/strength ratio, and can act as reinforcement when the interface contact area is adequate [47,48]. Instead, with the addition of 3 wt.% of KMW, mechanical reinforcement does not occur properly. This behavior can be explained by the more marked plasticizing effect produced by the KMW as a consequence of the possible degradation of some phenolic compounds (with less –OH able to establish hydrogen bonding interaction between them) present in pristine yerba mate, due to the hydrothermal extraction process used to prepare the infusion. Thus, permitting better interaction between PLA and ATBC allowed for a higher elongation at break, in good accordance with the T*_g_* value, close to that of r3-PLA-ATBC (Table 2).

### 3.7. Release Studies and Antioxidant Ability

**Figure 7:**
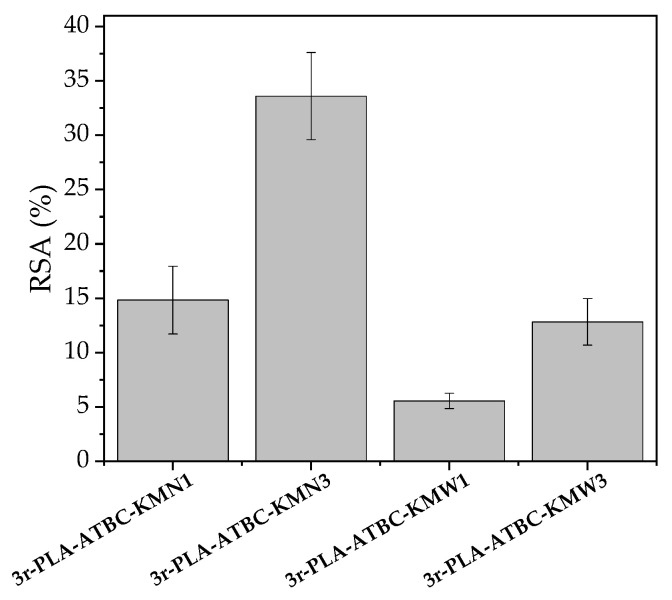
Radical scavenging activity of 3r-PLA-ATBC-based films.

Specific migration tests were conducted to evaluate the potential antioxidant activity of the films, as microbial cellulose obtained from kombucha fermentation showed that the tea used for its production had antioxidant properties [18]. In this work, kombucha was fermented in a yerba mate-sugared infusion. The antioxidant activity of yerba mate is well-known, and mainly arises from its composition in phenolic compounds [19]; even yerba mate waste is still able to provide antioxidant activity [15]. The radical scavenging activity (RSA) of each food simulant D1 sample after 10 days of contact at 40 °C, considered by the current legislation the worst foreseeable conditions for intended use [28], was determined by means of the DPPH method [41], and the results are shown in Figure 7.

As expected, the r3-PLA film did not show any antioxidant activity. The KMN and KMW were processed into thin films and also subjected to the food simulant for comparison. The neat KMN film showed high RSA activity of 70.2 ± 1.1% and 55.2 ± 11.7%. Meanwhile, the films loaded with KMN or KMW showed some antioxidant activity, as microbial cellulose obtained from kombucha fermentation possesses natural and remarkable antioxidant activity, which is directly related to the infusion used for the fermentation [18]. Films loaded with KMN showed higher antioxidant activity than those loaded with KMW, as it is known that yerba mate waste possesses a low polyphenol content due to the hydrothermal extraction process used during infusion preparation (temperature higher than 80 °C) before obtaining the waste. The scavenging effect obtained here for 3r-PLA-ATBC-based materials loaded with microbial cellulose kombucha fermented in yerba mate or yerba mate waste (between 1 wt.% and 3 wt.%) are low, but it should be highlighted that they still possess some antioxidant activity and, thus, the results interesting for food crops and food packaging. A high level of antioxidant activity has been observed in materials containing yerba mate extract. For instance, Deladino et al. studied corn starch-loaded materials with yerba mate extract at a concentration of around 10 wt.% with respect to the starchy matrix, and found between 40% and 60% RSA [19]. The values obtained in the present work are in the range of other antioxidant materials based on tri-layer recycled PLA/sodium caseinate (SC)/recycled PLA-based materials reinforced with 1 and 3 wt.% of nanoparticles obtained from yerba mate waste (YMN) (RSA (%) of rPLA/SC/rPLA-YMN1 = 6.4 ± 0.1 and RSA (%) of rPLA/SC/rPLA-YMN3 = 11.0 ± 0.2) [39].

Besides the biobased origin, biodegradability, and recyclability of PLA, the modification of PLA-based composite films through the incorporation of cellulosic nanoparticles has already shown improvements in thermal, barrier, and mechanical properties, and the possibility to provide additional antioxidant properties makes these films highly interesting for food packaging or agricultural applications [15,49].

### 3.8. Water Contact Angle and Water Vapor Transmission Rate

**Figure 8:**
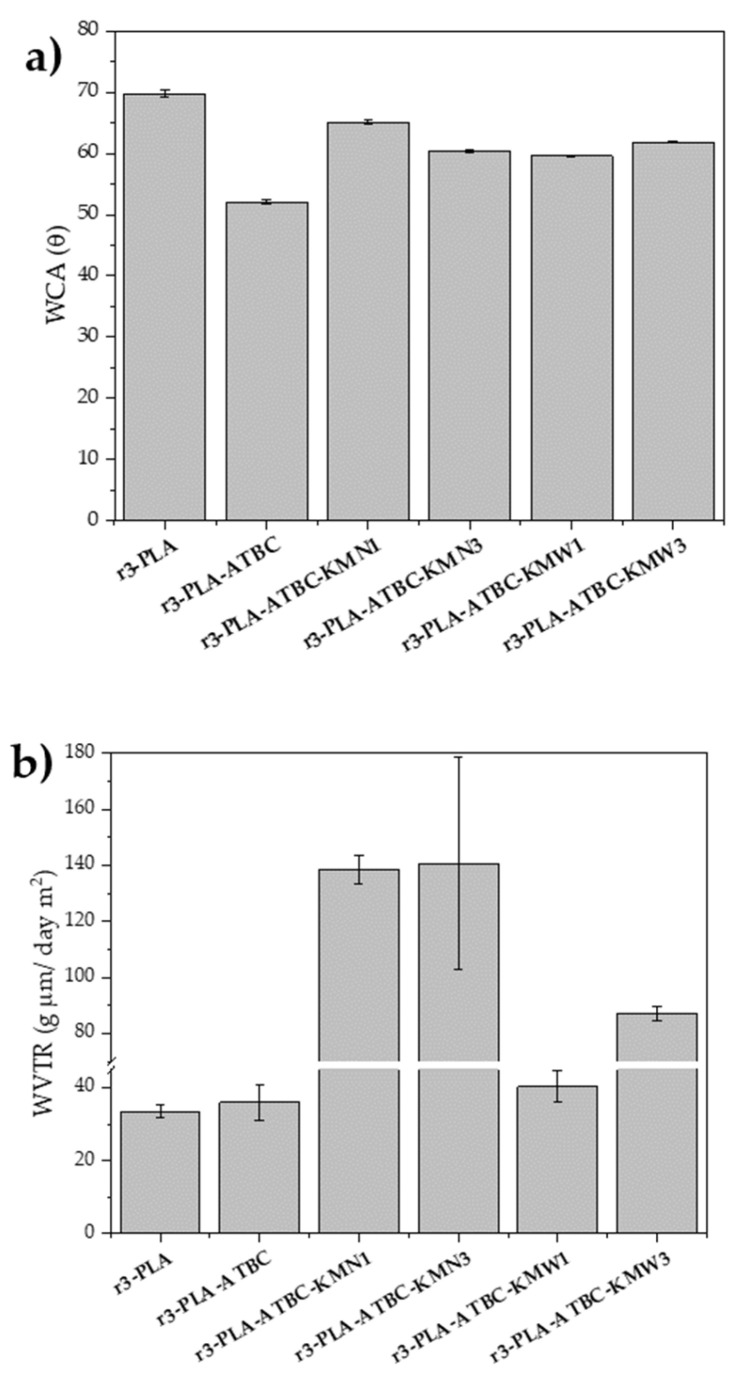
(**a**) Static water contact angle measurements and (**b**) water vapor transmission rate of 3r-PLA-ATBC-based films.

The surface hydrophilic/hydrophobic properties of films were determined by the measurement of the static water contact angle (WCA) and the results are reported in Figure 8a. Meanwhile, the water vapor transmission rate (WVTR) values of plasticized r3-PLA-ATBC-based films are reported in Figure 8b. The PLA film, after three cycles of melt extrusion (3r-PLA), showed a water contact angle higher than 65°, which was ascribed to hydrophobic surfaces (θ lower than 65° are ascribed to hydrophilic surfaces) [50]. The plasticized 3r-PLA sample (3r-PLA-ATBC) showed a lower WCA value, as was observed in an already reported work on PLA and PLA-ATBC [25]. This was also in agreement with the WVTR value, in which r3-PLA andr3-PLA-ATBC showed similar WVTR properties, despite being slightly higher the WVTR of 3r-PLA-ATBC. The plasticizing effect of ATBC into the r3-PLA matrix influenced the diffusion process as a consequence of the increased polymer chain mobility. The presence of either KMN or KMW in the 3r-PLA-ATBC matrix produced a decrease in the surface wettability of the films (Figure 8a), leading to values higher than 3r-PLA-ATBC and, thus, higher hydrophobicity. This unexpected increment of the hydrophobicity of the film surface, even when hydrophilic cellulosic particles were added, can be related to the changes in the topographical properties as a consequence of the presence of cellulose particles. However, it should be highlighted that the composite films were still more hydrophilic than 3r-PLA. The WVTR showed increased values with the presence of either KMN or KMW in the 3r-PLA-ATBC matrix (Figure 8b), particularly in the case of KMN, probably due to the high amount of active compounds.–OH groups were able to interact with water, increasing the water diffusion through the film. Meanwhile, the KMW, which showed less antioxidant activity (Figure 7) and, consequently, a lower amount of bioactive compounds within the polymeric matrix, allowed less water vapor to be transmitted through the film.

## 4. Conclusions

Microbial cellulose particles were successfully obtained from kombucha beverage fermented in both infusion, pristine yerba mate, and yerba mate waste. They were further used to reinforce a plasticized PLA matrix subjected to three extrusion cycles (r3-PLA), aiming to simulate the revalorization of PLA from industrial PLA products rejected during the production line. The r3-PLA-based biocomposites, reinforced with KMN and KMW, were effectively prepared by solvent casting, and the effect of yerba mate starting material (pristine or waste), as well as the amount added (1 and 3 wt.%) into the plasticized r3-PLA-ATBC, were deeply investigated.

All films resulted to be optically transparent, and FESEM micrographs revealed a good dispersion of microbial cellulose particles in the reprocessed polymeric matrix.

DSC analysis showed a crystallinity increase in r3-PLA-ATBC composites reinforced with KMW, indicating that it favored the crystal growth and nucleation effects, while for the tensile test, measurements showed a more marked plasticization effect. Moreover, the materials reinforced with KMW showed less WVTR, indicating improved barrier properties against water than the materials reinforced with pristine KMN. However, KMN-reinforced r3-PLA-ATBC-KMN-based films showed higher antioxidant activity, although it should be highlighted that r3-PLA-ATBC-KMW-based films still showed antioxidant activity.

The reprocessed PLA (r3-PLA) in combination with ATBC and KM particles offers a promising perspective to produce transparent and flexible films with good water barriers and mechanical properties that are suitable as antioxidant films for food packaging or agricultural mulch films.

## Figures and Tables

**Table 1:** Film formulations based on plasticized 3r-PLA-ATBC.

Sample	r3-PLA (wt.%)	ATBC (wt.%)	KMN (wt.%)	KMW (wt.%)
**r3-PLA**	100	-	-	-
**r3-PLA-ATBC**	85	15	-	-
**r3-PLA-ATBC-KMN1**	84.15	14.85	1	-
**r3-PLA-ATBC-KMN3**	82.45	14.55	3	-
**r3-PLA-ATBC-KMW1**	84.15	14.85	-	1
**r3-PLA-ATBC-KMW3**	82.45	14.55	-	3

**Table 2:** DSC thermal properties of 3r-PLA-ATBC-based films.

Sample	T*_g_* (°C)	T*_cc_* (°C)	Δ*H_cc_* (J g^−1^)	T*_mI_* (°C)	T*_mII_* (°C)	Δ*H_m_* (J g^−1^)	*χ_c_* (%)
**r3-PLA**	49.1	106.1	20.0	144.4	151.3	23.2	3.4
**r3-PLA-ATBC**	32.1	95.0	21.2	135.6	146.8	23.4	2.8
**r3-PLA-ATBC-KMN1**	39.1	99.5	19.2	139.7	148.9	19.8	0.7
**r3-PLA-ATBC-KMN3**	36.8	99.9	19.5	138.3	148.2	20.2	1.0
**r3-PLA-ATBC-KMW1**	37.3	98.4	20.3	137.7	148.1	22.8	3.1
**r3-PLA-ATBC-KMW3**	33.5	99.3	19.0	138.3	147.8	21.5	3.2

**Table 3:** TGA thermal properties of 3r-PLA-ATBC-based films.

Sample	T_5%_ (°C)	T_10%_(°C)	T_max_ (°C)	Residual Mass (%)
**r3-PLA**	296.1	313.52	354.4	0.4
**r3-PLA-ATBC**	217.4	272.28	351.1	0.8
**r3-PLA-ATBC-KMN1**	224.8	266.74	354.1	0.5
**r3-PLA-ATBC-KMN3**	214.7	246.21	344.7	0.7
**r3-PLA-ATBC-KMW1**	227.2	274.62	352.4	0.5
**r3-PLA-ATBC-KMW3**	208.1	243.19	351.8	0.5

## Data Availability

The data presented in this study are available on request from the corresponding author.
